# Contributions to Aural Medicine, with 19 Statistical Tables

**Published:** 1846-07

**Authors:** 


Beitrage zur Ohrenheilkunde. Von Dr. *Wilhelm Kramer.* Nebst 19 Statitischen Tabellen. 8vo. pp. 314. Berlin, 1845.

Contributions to Aural Medicine, with 19 Statistical Tables. By Dr. *William Kramer.*

The name of Dr. Kramer is well known in this country as the author of an excellent treatise on Diseases of the Ear, published some years ago, and which was translated by Dr. Bennett. We reviewed the translation at considerable length, and reported favourably of it at the time of its publication—vide Med. Chir. Rev. for July 1838. The present work consists chiefly of statistical tables and illustrations that serve to confirm the leading positions in his former one. The other articles are a paper on the Acoustics of the Human Ear, one on the application of Electro-Magnetism in the Treatment of Deafness, and one on Cerebral Otorrhœa. The writings of Dr. Kramer everywhere exhibit the spirit of an enlightened and judicious author, in whose statements we can place perfect reliance. Would that we could say the same of most aurists in this country! There is no branch of the healing art in which charlatanery is more rampant than in aural surgery, among those, at least, who profess to be ear-doctors. In short, they are little better than advertising quacks.

